# Comparative Safety Profiles of Sedatives Commonly Used in Clinical Practice: A 10-Year Nationwide Pharmacovigilance Study in Korea

**DOI:** 10.3390/ph14080783

**Published:** 2021-08-09

**Authors:** Yeo-Jin Choi, Seung-Won Yang, Won-Gun Kwack, Jun-Kyu Lee, Tae-Hee Lee, Jae-Yong Jang, Eun-Kyoung Chung

**Affiliations:** 1 Department of Clinical Pharmacy, Graduate School of Clinical Pharmacy, CHA University, Seongnam 13488, Korea; yjchoi@cha.ac.kr; 2 Department of Pharmacy and Yonsei Institute of Pharmaceutical Sciences, College of Pharmacy, Yonsei University, Incheon 21983, Korea; syang345@gmail.com; 3 Division of Pulmonary, Allergy and Critical Care Medicine, Kyung Hee University Hospital, Seoul 02447, Korea; wongunnim@naver.com; 4 Department of Internal Medicine, Dongguk University Ilsan Hospital, Goyang 10326, Korea; gerome@dumc.or.kr; 5 Institute for Digestive Research, Soonchunhyang University Seoul Hospital, Seoul 04401, Korea; iman0825@naver.com; 6 Department of Internal Medicine, Kyung Hee University College of Medicine, Seoul 02447, Korea; jyjang@khu.ac.kr; 7 Department of Pharmacy, College of Pharmacy, Kyung Hee University, Seoul 02447, Korea; 8 Department of Pharmacy, Kyung Hee University Hospital at Gangdong, Seoul 05278, Korea

**Keywords:** sedatives, drug safety, patient safety, adverse events, pharmacovigilance, KAERS

## Abstract

This study aims to compare the prevalence and seriousness of adverse events (AEs) among sedatives used in critically ill patients or patients undergoing invasive procedures and to identify factors associated with serious AEs. Retrospective cross-sectional analysis of sedative-related AEs voluntarily reported to the Korea Adverse Event Reporting System from 2008 to 2017 was performed. All AEs were grouped using preferred terms and System Organ Classes per the World Health Organization—Adverse Reaction Terminology. Logistic regression was performed to identify factors associated with serious events. Among 95,188 AEs, including 3132 (3.3%) serious events, the most common etiologic sedative was fentanyl (58.8%), followed by pethidine (25.9%). Gastrointestinal disorders (54.2%) were the most frequent AEs. The most common serious AE was heart rate/rhythm disorders (33.1%). Serious AEs were significantly associated with male sex; pediatrics; etiologic sedative with etomidate at the highest risk, followed by dexmedetomidine, ketamine, and propofol; polypharmacy; combined sedative use; and concurrent use of corticosteroids, aspirin, neuromuscular blockers, and antihistamines (reporting odds ratio > 1, *p* < 0.001 for all). Sedative-induced AEs are most frequently reported with fentanyl, primarily manifesting as gastrointestinal disorders. Etomidate is associated with the highest risk of serious AEs, with the most common serious events being heart rate/rhythm disorders.

## 1. Introduction

Sedation is critical in minimizing discomforts in patients, especially in those undergoing invasive procedures such as gastrointestinal endoscopy or hospitalized in an intensive care unit (ICU) [1,2]. Sedation differs from general anesthesia in a way that sedated patients have limited response to repeated external stimuli, typically without impairment of cardiovascular functions, while individuals under general anesthesia completely lose response to any level of stimuli, potentially with impaired cardiovascular functions [3]. Sedation is stratified into minimal (anxiolysis), moderate, and deep sedation [3]. Proper sedation not only relieves discomforts but also improves stress responses, anxiety, and patient outcomes; however, the debate on the appropriate depth of sedation in patients requiring critical care or invasive procedures is still ongoing [1,2,3,4]. Indeed, some previous studies suggested inappropriate sedation, particularly oversedation, which frequently occurs in clinical practice, may increase the duration of mechanical ventilation, hospital or ICU stays, and in-hospital mortality [4,5,6].

Despite the importance of appropriate depth of sedation, optimal sedative drug therapy is still a challenge in clinical practice and differs based on the purpose of sedation [1,7]. The current guideline for the management of pain, agitation/sedation, delirium, immobility, and sleep disruption (PADIS), published by the Society of Critical Care Medicine (SCCM), strongly recommends non-benzodiazepine sedatives such as propofol or dexmedetomidine over benzodiazepines for inducing sedation in critically ill patients including those who require mechanical ventilation because of benzodiazepine-related adverse events (AEs) [8]. Although benzodiazepines had been the primary sedative agents previously and still continue to be prescribed commonly in clinical practice, their use has declined over the last decade mainly due to their AEs associated with the central nervous system, including impaired memory, attention, learning, or visuospatial abilities [9]. These benzodiazepine-induced AEs are associated with substantial morbidity and mortality, such as benzodiazepine-related fall injury in elderly patients [9]. Therefore, the SCCM recommends alternatives to benzodiazepines as the preferred sedative agent in the PADIS guideline [8]. However, deviations from this guideline have been reported in the actually prescribed sedative therapy, indicating potential misuse of sedatives [10,11,12]. Actually, the most frequently prescribed sedatives in Korea included benzodiazepines (diazepam, midazolam); non-benzodiazepines including propofol, dexmedetomidine, etomidate, ketamine, opioids (e.g., alfentanil, fentanyl, remifentanil), and thiopental; and the combined use of two or more different sedatives [10,13,14]. However, the safety profile of these sedative agents, including their combined use, is yet to be comparatively evaluated despite the increasing use of sedatives.

Sedatives are implicated in various adverse drug events (ADEs), ranging from common, mild toxicities such as gastrointestinal AEs to more serious toxicities, including cardiac and respiratory complications [12,15,16]. These ADEs limit the use of sedatives in clinical practice, which may be aggravated by inconsiderate sedative use and habitual prescribing patterns, including combined sedative use [12,15]. However, each sedative agent has a highly variable pattern of ADEs, including the type and seriousness of sedative-related AEs, which makes the management and prevention of sedative-induced AEs even more challenging in clinical practice [15,17]. With the variable demographic, clinical, and sociocultural aspects associated with medication use in clinical practice, assessment of the real-world data for sedative use in a specific country and/or ethnic group may help promote safe and effective use of sedatives [18]. Because Korea has the National Health Insurance (NHI) system, the NHI service sample (NHISS) cohort is usually the most commonly used real-world data source. However, the NHISS cohort does not contain non-reimbursement data records. Considering the majority of sedatives are prescribed without insurance reimbursement in Korea [19], the NHISS cohort may not be the best data source to show the real-world pattern of sedative usage and safety; rather, voluntary AE reports may be more appropriate because (1) they include AEs associated with both non-reimbursed and reimbursed medications and (2) healthcare practitioners are legally required to report clinically significant AEs. Therefore, the aims of this study were to compare the prevalence and seriousness of ADEs associated with sedatives commonly used for invasive procedures or the management of critically ill patients in Korea and to identify factors associated with serious sedative-related ADEs utilizing spontaneous AE reporting data.

## 2. Results

### 2.1. Baseline Demographics

**Figure 1:**
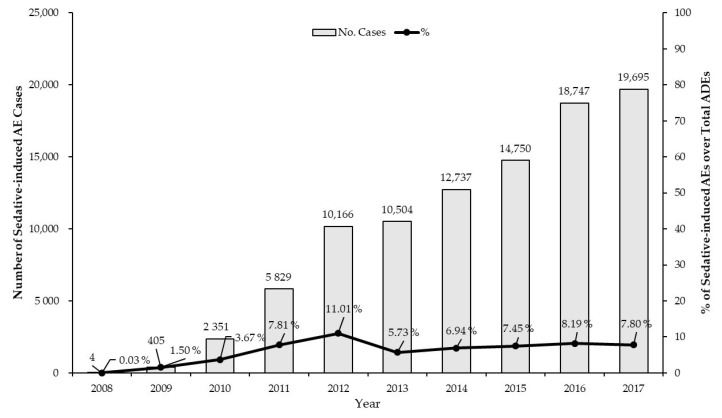
The number and rate of sedative-induced adverse events (AEs) reported from 2008 to 2017 (ADEs, adverse drug events).

Overall, 95,188 sedative-related ADEs in 68,656 patients from January 2008 to December 2017 were identified from the Korea Adverse Event Reporting System (KAERS). The number of reported ADE cases has substantially increased every year, with a relatively constant rate of sedative-induced ADEs at approximately 10% or less of all ADE reports submitted to the KAERS over the study period (Figure 1). Among the causative agents included in this analysis, fentanyl was the most common etiologic agent (n = 55,964; 58.8%), followed by pethidine (n = 24,668; 25.9%) (Table 1). Serious AEs occurred in 3132 cases (3.3%) most frequently associated with pethidine (n = 1019; 31.5%), followed by fentanyl (n = 819; 25.3%) and midazolam (n = 497; 15.4%). The median duration of therapy was <1 day for all sedatives included in the analysis, with the maximum duration of treatment being 31 days until the onset of AEs. The baseline demographic characteristics of sedative-related ADE cases are summarized in Table 2. The majority of sedative-induced ADE cases occurred in females (n = 62,798; 66.6%) and individuals with advanced age of 50 years and older (n = 52,450; 55.1%).

### 2.2. Types of ADEs Induced by Sedatives

Sedative-induced ADEs based on the System Organ Classes (SOC) classification system are presented in Table 3. Overall, the most common ADEs associated with sedative use were gastrointestinal disorders (n = 51,577; 54.2%), including nausea and vomiting, followed by nervous system disorders (n = 23,833; 25.0%) such as decreased consciousness and dizziness. The majority of sedative-related, nonserious ADEs were gastrointestinal disorders (n = 51,317 (55.8%)) and nervous system disorders (n = 23,363 (25.4%)) (Table 3). Among sedative-induced serious AEs (n = 3132; 3.3%), heart rate and rhythm disorders (n = 1038; 33.1%) including, hypotension, most frequently occurred, followed by respiratory system disorders (n = 608; 19.4%) such as dyspnea and reduced oxygen saturation; both heart rate/rhythm disorders and respiratory system disorders were more likely to be serious events (ROR = 16.724 and 22.046, respectively (95% CIs 15.379–18.187 and 19.782–24.569, respectively); *p* < 0.001 for both). Death occurred in 61 cases (0.06%), primarily associated with propofol (n = 15), midazolam (n = 14), and diazepam (n = 12). No fatal cases were reported with alfentanil, etomidate, sufentanil, and thiopental.

### 2.3. Factors Associated with Seriousness of Sedative-Related ADEs

In the univariate analysis, the seriousness of sedative-related AEs was significantly associated with sex, age, causality, etiologic sedative agent, the number of concurrently used medications, and the concomitantly used medication (*p* < 0.001, Table 4). All of the investigated factors remained significantly associated with the seriousness of sedative-induced AEs in the multivariate regression model (*p* < 0.001, Table 4). Serious AEs were more likely to be reported in the followings: male patients; patients younger than 10 years old; patients treated with dexmedetomidine, diazepam, etomidate, ketamine, midazolam, pethidine, propofol, remifentanil, and thiopental; and those taking multiple medications (*p* < 0.001 for all). Among the tested sedatives, etomidate was associated with the highest risk for developing serious AEs (OR 52.232 (95% CI 15.943–171.118)), followed by dexmedetomidine (OR 35.784 (95% CI 11.120–115.153)), ketamine (OR 31.322 (95% CI 9.865–99.453)), and propofol (OR 27.226 (95% CI 8.669–85.504)). Serious sedative-induced AEs were more likely to occur in patients treated with a sedative in combination with corticosteroids (OR 2.933 (95% CI 2.187–3.935)), aspirin (OR 2.900 (95% CI 1.771–4.750)), benzodiazepines (OR 2.370 (95% CI 1.703–3.299)), neuromuscular blockers (OR 1.640 (95% CI 1.086–1.712)), antihistamines (OR 1.557 (95% CI 1.096–2.212)), and another sedative (OR 1.272 (95% CI 1.121–1.444)). In contrast, patients concurrently treated with a non-steroidal anti-inflammatory drug (NSAID), an opioid analgesic, a serotonin (5HT_3_) antagonist, or zolpidem were less likely to develop serious sedative-related AEs.

## 3. Discussion

The global prevalence of sedative use is increasing every year, particularly in Korea [20]. In 2019, the Ministry of Food and Drug Safety (MFDS) in Korea reported controlled substances were administered to more than 36% of the total Korean population (18,502,227 Koreans), most frequently propofol (n = 8,505,249), followed by midazolam (n = 6,583,018), diazepam (n = 3,248,581), meperidine (i.e., pethidine; n = 2,476,722), and fentanyl (n = 1,905,655) [21]. Although the majority of these sedative agents were recommended to be used by physicians with anesthetic training, approximately 60% of the sedative prescriptions were written by primary care physicians (PCPs) without adequate anesthetic training [21,22]. Although single-dose or short-term sedative therapy might be generally perceived to be safe among PCPs, the median time elapsed between the start of sedative therapy and the development of sedative-induced AEs was less than 1 day in our present study (Table 2), suggesting a substantial risk of AEs posed by short-term, acute pharmacologic sedation [23]. Moreover, evidence-based practice of acute, short-term sedation therapy for patients undergoing invasive procedures or hospitalized in an ICU is even more challenging due to the lack of clinical practice guidelines based on comprehensive scientific data, leading to high variability in sedative use in the real-world setting [2,12]. In fact, our current study suggested a high prevalence of sedative administration concomitantly with other sedatives or other medications with sedation side effects, resulting in a significantly increased risk of developing serious AEs (Table 4). To the best of our knowledge, the safety profile of sedatives commonly used in clinical practice has been comparatively assessed in only a few head-to-head studies or literature reviews [24,25,26,27]. However, even the recent head-to-head studies were not statistically powered for safety endpoints [26,27]. In addition, one of the head-to-head studies was a prospective trial conducted in a controlled environment, suggesting limited generalizability of the trial results to the real-world practice setting [27]. Therefore, our present study may substantially contribute to the current body of literature by characterizing the prevalence and pattern of sedative-induced AEs as well as identifying factors associated with serious sedative-related AEs in Korea through comprehensively analyzing the real-world spontaneous AE reports of sedatives commonly used for invasive procedures or critical care in Korea.

In this study, the most frequently reported causative agent associated with sedative-induced AEs was fentanyl, followed by pethidine, accounting for approximately 85% of all sedative-related AEs (Table 1). Although the true risk of developing sedative-induced AEs might be higher for fentanyl and pethidine compared to other sedatives, caution should be exercised when interpreting the reported incidence of AEs in our present study. The reported ADE incidence might be confounded by the amount of each sedative use, potentially leaving the true relative risk of developing ADEs associated with each sedative unclear. In fact, drug utilization data from the MFDS showed fentanyl (n = 1,905,655) and pethidine (n = 2,476,722) were among the most commonly used sedative agents in Korea [21]. Considering frequent non-reimbursed use of sedatives in clinical practice in Korea, the actual use of sedatives might be much greater in the real-world setting, possibly resulting in a high prevalence of reported AEs associated with fentanyl and pethidine [28]. However, as shown in Table 4, pethidine was associated with a significantly increased risk of serious AEs, suggesting the true risk of pethidine-induced toxicity might be high. Future studies are warranted to estimate the relative risk of developing AEs associated with each sedative use while adjusting for the prevalence of their use.

Our present study suggested gastrointestinal disorders such as nausea and vomiting and nervous system disorders including decreased consciousness and dizziness as the most common sedative-induced AEs, accounting for approximately 80% of all sedative-related AEs (Table 3). Our study findings were largely consistent with previous studies reporting nervous system suppression, nausea, and vomiting as primary AEs associated with sedatives [29]. The majority of gastrointestinal and nervous system disorders induced by sedative use were nonserious, mild events. However, serious events did occur in 0.50% and 1.97% of all gastrointestinal and nervous system AEs, respectively, highlighting the importance of adequate patient education regarding these AEs and thorough monitoring for early detection and appropriate management.

In our current study, serious events accounted for 3.3% (n = 3132) of the total sedative-induced AEs, suggesting a substantially lower reporting rate of serious events compared to that in the United States (16.9%) [30]. This might be associated with the reporting pattern of ADEs in Korea [31]. Although the spontaneous AE reporting systems have been primarily used to report major serious AEs or rare reactions for signal detection and discovery of potential ADEs [32], our study showed frequent reporting of common, nonserious sedative-related AEs, potentially resulting in the apparently lower risk of serious events. Among the sedative agents investigated in this study, pethidine was most frequently associated with serious AEs (Table 1), implying a higher risk for pethidine to develop serious AEs. In fact, pethidine has been notorious for its relatively high risk of serious central nervous system AEs including tremors, muscle twitches, hyperactive reflexes, agitation, and seizures owing to its active metabolite, normeperidine [14,33]. However, caution should be exercised when interpreting the risk of pethidine for serious AEs because, as aforementioned, the high reporting frequency of pethidine-induced serious AEs might result from its prevalent use in Korea [21]. Similarly, although fentanyl was the second most commonly reported etiologic agent of serious sedative-related AEs, it might be associated with the high prevalence of its use, considering the relative reporting frequency of fentanyl-induced serious AE cases compared to the total AE cases caused by fentanyl (Table 1). The major sedative-induced serious AEs based on the SOC disorders was heart rate and rhythm disorders such as hypotension (33.1%), followed by respiratory system disorders such as dyspnea (19.4%) and nervous system disorders such as decreased consciousness and dizziness (15.0%), accounting for at least two-thirds of serious sedative-related AEs (Table 4). Among them, respiratory system disorders, as well as heart rate and rhythm disorders, were more likely to be serious (ROR 22.046 (95% CI 19.782–24.569) for respiratory system disorders; ROR 16.724 (95% CI 15.379–18.187) for heart rate and rhythm disorders); in contrast, nervous system disorders were less likely to be serious (ROR 0.519 (95% CI 0.470–0.573)). Our findings were consistent with a previous study analyzing AEs associated with procedural sedatives [34]. Similar to our current study, Mason et al. suggested that all reported sedative-induced cardiovascular and respiratory AEs were moderate to sentinel events; however, most of the neuropsychiatric and gastrointestinal AEs caused by procedural sedatives were minimal to minor events [34]. Considering a number of cardiovascular and respiratory AEs are listed as potentially serious AEs in the prescribing information for commonly used sedatives and often occur unexpectedly [35,36,37,38], close monitoring systems must be in place for prompt and appropriate management of sedative-induced AEs, particularly in patients at an increased risk for developing serious AEs associated with sedative use.

Based on the univariate and multivariate analyses, our present study suggested factors significantly increasing the risk of developing serious sedative-induced AEs (Table 4). Similar to previous studies, serious AEs were more likely to be reported in males, patients at the extremes of age (i.e., younger than 10 years old (Table 4), patients at the age of 60 or greater (OR = 1.987, 95% CI 1.586–2.201 when compared to those 20 to 29 years old; data not shown)), and those taking multiple medications [39,40]. Notably, our study comparatively evaluated the risk of etiologic sedatives for developing serious AEs as well as the impact of concomitant medications on the risk of serious AEs (Table 4). Compared to alfentanil with the lowest relative frequency of sedative-related serious AEs reported in our study (Table 1), the risk of developing serious AEs was 52-, 35-, 31-, and 27-fold higher for etomidate, dexmedetomidine, ketamine, and propofol, respectively (Table 4); only fentanyl and sufentanil showed a comparable risk of developing sedative-induced serious AEs to alfentanil (*p* > 0.05). Although the risk of etomidate, dexmedetomidine, and ketamine for developing serious AEs, including cardiovascular and/or respiratory toxicities, might be truly higher than that of other sedatives assessed in this study [30,41], their frequent use as an adjunct therapy to other primary sedatives such as fentanyl, propofol, and benzodiazepines might contribute to the significantly increased risk for serious AEs as shown in this study (Table 4). Of our great concern was the high risk of propofol for serious AEs considering the prevalent use of propofol for sedation in patients undergoing invasive procedures such as endoscopy and hospitalized in an ICU [29]. Previous studies suggested propofol as a relatively safe sedative, used alone or in combination with other adjunctive sedatives, with a lower incidence of AEs for invasive procedures and critical care [8,30,42]. Consequently, propofol has been commonly used with or without anesthesiologist involvement in various settings of clinical practice, including outpatient endoscopic procedures in primary care clinics in Korea [21,22,43]. Because propofol is one of the primary sedative agents possibly used in combination with other adjunct therapy [44], the propofol-based combinational sedation therapy might contribute to the increased risk for serious propofol-related AEs (Table 4). However, the significant increase in the risk for serious AEs was not observed for fentanyl (*p* > 0.05), which is another primary sedative in a comparable therapeutic place to propofol (Table 4), suggesting a truly high risk of propofol for serious sedative-induced AEs such as cardiovascular events. In fact, similar to our current study, a previous study conducted by Duprey and colleagues reported an increased risk of serious cardiovascular AEs associated with propofol compared to other sedatives [30].

Among the concomitantly used medications other than sedatives, our study suggested a significantly increased risk of serious sedative-induced AEs in patients concurrently receiving corticosteroids, aspirin, neuromuscular blockers, or antihistamines; however, the risk of serious AEs was significantly lower in those co-administered NSAIDs, opioid analgesics, or serotonin (5-HT_3_) antagonists (Table 4). The higher likelihood of developing serious AEs in patients concomitantly receiving corticosteroids and neuromuscular blockers might be associated with the severity of underlying diseases. Current clinical practice guidelines suggested the scheduled use of corticosteroids for the treatment of critical illness-related corticosteroid insufficiency commonly occurring in critically ill patients, including those with severe bacterial infections (e.g., pneumonia, meningitis), undergoing cardiopulmonary bypass surgery, and suffering a cardiac arrest [45,46]. Neuromuscular blocking agents may be used for facilitating endotracheal intubation, optimizing mechanical ventilation, and managing other life-threatening conditions in critically ill patients [47,48]. Considering these critically ill patients commonly have respiratory distress and hemodynamic instability [49], the severe underlying disease rather than the concomitant use of corticosteroids and neuromuscular blockers itself might contribute to the common serious sedative-related AEs of heart rate/rhythm and respiratory system disorders. Similarly, the increased risk of sedative-related serious AEs in patients concurrently taking aspirin might be associated with their underlying cardiovascular diseases requiring aspirin therapy (e.g., ischemic heart disease, myocardial infarction), potentially resulting in serious hemodynamic complications such as hypotension (Table 4). The concomitant use of antihistamines (H_1_ antagonists) was significantly associated with an increased risk of serious sedative-induced AEs due to additive and/or synergistic sedative effects, potentially resulting in oversedation. In contrast, serious events caused by sedative agents were less likely to occur in patients concomitantly receiving analgesic therapy, including NSAIDs and opioids (Table 4). Optimal pain control with adequate analgesic therapy reduces the risk of pain-related agitation [8,49]; thus, excessive use of sedatives may not be necessary for patients concurrently treated with analgesics as well as sedatives, minimizing the risk of oversedation through achieving adequate pain control. Therefore, the risk of serious sedative-related AEs commonly associated with oversedation is substantially lower in these patients. Consistently, our current study suggests a lower risk of serious AEs caused by sedatives when used in combination with 5-HT_3_ antagonists due to their anti-nausea effects contributing to a decrease in the perception of noxious stimuli. Ultimately, agitation is less likely and can be managed without excessive sedation in these patients, leading to fewer serious sedative-related AEs. Additional future studies are warranted to examine the effects of concomitant medications on the risk of developing serious sedative-related AEs, adjusted for other confounding risk factors such as the sedative agent used in the patient and the severity of underlying diseases.

This study has several limitations that need to be acknowledged. First, because the KAERS database is a spontaneous reporting system, caution should be exercised when interpreting our study results due to possible under-reporting of AEs and substantial missing information such as indications, comorbid conditions, and concurrent medication therapy. Second, inter-reporter variability might influence the quality of the recorded data in the KAERS database. For example, although the seriousness of the reported ADEs was evaluated based on the ICH guideline definition, the individual subjective judgment might contribute to determining whether a specific ADE case was serious or not. In addition, potential reporting bias might exist, which could result in selective over-reporting of some ADEs and under-reporting of others [31]. Nevertheless, the majority of ADE cases, particularly those induced by sedatives, were reported by healthcare professionals (Table 2) [50], suggesting adequate reliability and appropriateness of the recorded ADE data. Furthermore, the number of ADE reports for a specific medication can be influenced by the prevalence of using the medication in clinical practice. Thus, our current study findings should be carefully interpreted. Lastly, this study should not be translated to determine a definite causal relationship between the reported ADE and the offending sedative owing to the nature of the study design. Despite the verification of the causality by a reporter and an independent record reviewer, nondrug factors or other medications than the reported culprit sedative might result in the recorded AE. Therefore, larger-scale clinical studies may be warranted to evaluate the effects of a specific sedative medication on the risk of the reported ADEs, taking into account potential confounding variables such as concomitant medications and underlying diseases.

## 4. Materials and Methods

### 4.1. Data Source and Definitions

This was a retrospective, cross-sectional study analyzing ADE records spontaneously reported from January 2008 to December 2017 to the KAERS. The KAERS is a voluntary, spontaneous ADE reporting system, constructed by the Korea Institute of Drug Safety and Risk Management (KIDS), available to the public as well as healthcare professionals [18,51]. When clinically significant ADE cases occurred in clinical practice, they were reported by the healthcare professionals or the general public using the structured ADE case reporting form developed and validated by the KIDS, which is the national pharmacovigilance agency in Korea [51,52]. The ADE case reports submitted to the KAERS were reviewed and further verified by healthcare professional in the KIDS to minimize biases [18,51]. The ADE case reporting data, collected and validated by several individuals, were then deposited into the KAERS [18].

In the current analysis, included cases were ADEs reportedly caused by the following sedative agents commonly used for invasive procedures or management of critically ill patients in Korea [10,13,14]: alfentanil, dexmedetomidine, diazepam, etomidate, fentanyl, ketamine, midazolam, pethidine (meperidine), propofol, remifentanil, sufentanil, and thiopental. The following information was extracted from the KAERS database: (1) patient demographics including age, sex, and comorbidities; (2) medication information including concomitant medications, dates of treatment initiation and cessation, and the causative agent; (3) ADE information including the year of ADE reports and causality assessment between the reported ADE and the offending agent; and (4) seriousness of ADEs [18].

Causality of each ADE report was initially determined by the reporting individual and later verified by healthcare professionals in the KIDS based on the World Health Organization—Uppsala Monitoring Centre (WHO-UMC) criteria as follows: (1) certain, (2) probable/likely, (3) possible, (4) unlikely, (5) conditional/unclassified, and (6) unassessable/unclassifiable [53]. Adverse drug event records with causality determined as “certain”, “probable/likely”, and “possible” were included in the present analysis as sedative-related ADEs [51]. All ADEs were reported in preferred terms or included terms according to the WHO—Adverse Reaction Terminology (WHO-ART) [54], and they were grouped into SOC disorders. When two or more ADEs were reported in the same patient, each event was analyzed as a separate case. The seriousness of each ADE was assessed based on the International Conference on Harmonization (ICH) E2D Guideline, where serious ADEs were defined as AEs leading to (1) death, (2) life-threatening conditions, (3) hospitalization or prolongation of existing hospitalization, (4) persistent or significant disability or incapacity, (5) congenital abnormalities or birth defects, and (6) other medically significant events [55].

The study protocol for utilizing the KAERS database was approved by the KIDS (No. 1807A0028) and the institutional review board of Dongguk University Ilsan Hospital (No. DUIH 2021-03-023) (Goyang, South Korea). Informed consents were exempted by the board.

### 4.2. Statistical Analysis

SPSS Statistics 25.0 (Version 25.0; IBM SPSS Statistics for Windows, Armonk, NY, USA) was utilized for all statistical analyses. Patient demographics and reported ADEs were summarized using descriptive statistics with either mean ± standard deviation or median and range for continuous variables based on the Kolmogorov–Smirnov normality test results. The association of the SOC-based ADEs with their seriousness was evaluated using disproportionality analysis by estimating the reporting odds ratios (RORs) with the corresponding 95% confidence intervals (CIs) and the Mantel–Haenszel adjusted *p* values [40,56,57]. Disproportionality was defined as the lower limit of the 95% CI greater than 1 or the upper limit of the 95% CI less than one [40,56,57]. The result was considered reliable if the number of reported cases was greater than four [40,56,57].

The univariate analysis was performed to identify factors associated with serious AEs, and factors explored in the univariate analysis included sex, age, causality (certain, probable/likely, or possible), etiologic sedative agent, the number of concurrently used medications, and the use of each concomitant medication. Multiple logistic regression using a stepwise forward method was performed by investigating the factors significantly associated with the seriousness of sedative-related ADEs in the univariate analysis. Variables with *p* value < 0.1 in the univariate analysis and clinical plausibility were assessed in multiple logistic regression. Any *p* value < 0.05 was considered statistically significant in the multivariate logistic regression analysis.

## 5. Conclusions

The prevalence of sedative-related ADEs has been increasing every year in Korea. The most frequent culprit drug of sedative-induced ADEs is fentanyl. The most common AEs caused by sedatives are gastrointestinal disorders such as nausea and vomiting, followed by nervous system disorders including impaired consciousness and dizziness, the majority of which are nonserious events. Serious events occur relatively commonly (approximately 3% of the total sedative-induced AEs), with pethidine reported as the primary etiologic agent of serious AEs. The most frequently documented serious events associated with sedative use are heart rate/rhythm disorders such as hypotension and respiratory system disorders, including dyspnea and reduced oxygen saturation. The risk for developing serious AEs may be different among sedatives; the risk is relatively high with etomidate, dexmedetomidine, ketamine, and propofol, while it is relatively low with alfentanil, fentanyl, and sufentanil. Other factors increasing the likelihood of serious AEs include male sex; extremes of age; polypharmacy; concurrent sedative use; and concomitant use of corticosteroids, aspirin, neuromuscular blockers, or antihistamines. Patients concurrently receiving NSAIDs, 5-HT_3_ antagonists, or opioid analgesics are at a lower risk for serious sedative-related AEs. Taking into account sedative treatment regimens, concomitant medications, and other patient characteristics, close monitoring for patients receiving sedation therapy is warranted to ensure patient safety in clinical practice.

## Figures and Tables

**Table 1:** Sedatives implicated in certain, probable/likely, and possible adverse event (AE) cases (reported frequency (%)).

	Serious AEs (n = 3132)	Nonserious AEs (n = 92,056)	Total AE Cases (n = 95,188)
Alfentanil	3 (0.1)	671 (0.7)	674 (0.7)
Dexmedetomidine	65 (2.1)	252 (0.3)	317 (0.3)
Diazepam	127 (4.1)	2214 (2.4)	2341 (2.5)
Etomidate	48 (1.5)	126 (0.1)	174 (0.2)
Fentanyl	809 (25.8)	55,155 (59.9)	55,964 (58.8)
Ketamine	141 (4.5)	585 (0.6)	726 (0.8)
Midazolam	484 (15.5)	4398 (4.8)	4882 (5.1)
Pethidine	996 (31.8)	23,672 (25.7)	24,668 (25.9)
Propofol	247 (7.9)	1509 (1.6)	1756 (1.8)
Remifentanil	186 (5.9)	2691 (2.9)	2877 (3.0)
Sufentanil	2 (0.1)	405 (0.4)	407 (0.4)
Thiopental	24 (0.8)	378 (0.4)	402 (0.4)

**Table 2:** Baseline demographic characteristics of patients from whom adverse events (AEs) caused by sedatives were reported to the KAERS (n = 95,188).

Characteristics	No. of Cases (% Relative Frequency) or Median [Range]
**Age ^a^ (Years)**	53 [0–115]
<10 years	1872 (2.0)
10–19 years	4648 (5.1)
20–29 years	7300 (8.0)
30–39 years	10,588 (11.5)
40–49 years	14,908 (16.2)
50–59 years	18,743 (20.4)
60 years or older	33,707 (36.7)
**Sex ^b^**	
Male	31,524 (33.4)
Female	62,798 (66.6)
**Causality ^c^**	
Certain	1822 (1.9)
Probable/likely	44,214 (46.5)
Possible	49,152 (51.6)
**Treatment Duration ^d^ (Days)**	0 (0–31)
<1 day	45,002 (47.3)
1 day	12,756 (13.4)
2 days	6780 (7.12)
3 days	2727 (2.86)
≥4 days	3590 (3.77)
**Seriousness**	
Serious events ^e^	3132 (3.3)
Nonserious events	92,056 (96.7)
**Individuals Reporting the Sedative-Induced AEs ^f^**	
Physicians	8367 (9.2)
Pharmacists	7066 (7.8)
Nurses	67,881 (75.0)
General Public	78 (0.1)
Others	7142 (7.90)
**No. of Medications Used**	
1	70,392 (74.0)
2	12,930 (13.6)
3	4994 (5.3)
4	1665 (1.8)
5	814 (0.9)
6	575 (0.6)
7	460 (0.5)
8	364 (0.4)
≥9	2994 (3.1)
**No. of Comorbidities ^g^**	
1	61,752 (95.8)
2	1779 (2.8)
3	543 (0.8)
4	160 (0.3)
≥5	237 (0.4)

^a^ Missing in 3422 (3.6%) cases. ^b^ Missing in 866 (0.9%) cases. ^c^ Causality was assessed according to the World Health Organization—Uppsala Monitoring Centre (WHO-UMC) criteria. ^d^ Missing in 24,333 (25.6%) cases. ^e^ Defined as those resulting in death, life-threatening conditions, hospitalization or prolongation of existing hospitalization, persistent or significant disability or incapacity, congenital abnormalities or birth defects, or any other medically significant events based on the International Conference on Harmonization (ICH) E2D Guidelines. ^f^ Missing in 4654 (4.9%) cases. ^g^ Missing in 30,717 (32.3%) case.

**Table 3:** System Organ Class-based adverse events (AEs) caused by sedatives.

SOC Disorder Class	Serious AEs (n = 3132)	Nonserious AEs (n = 92,056)	Total AEs (n = 95,188)	*p* Value ^a^	ROR (95% CI) ^b^
Skin and appendages	156 (5.0)	5532 (6.0)	5688 (6.0)	0.017	0.820 (0.696–0.965)
Musculoskeletal system	9 (0.3)	91 (0.1)	100 (0.1)	0.001	2.912 (1.467–5.782)
Collagen	0 (0.0)	2 (<0.1)	2 (<0.1)	N/E ^c^	N/E ^c^
Central and peripheral nervous system (e.g., decreased consciousness, dizziness)	470 (15.0)	23,363 (25.4)	23,833 (25.0)	<0.001	0.519 (0.470–0.573)
Vision	10 (0.3)	82 (0.1)	92 (0.1)	0.001	3.593 (1.962–6.934)
Hearing and vestibular	1 (< 0.1)	11 (<0.1)	12 (<0.1)	N/E ^c^	N/E ^c^
Special senses (other) ^d^	0 (0.0)	4 (<0.1)	4 (<0.1)	N/E ^c^	N/E ^c^
Psychiatric	143 (4.6)	3200 (3.5)	3343 (3.5)	0.001	1.328 (1.119–1.577)
Gastrointestinal (e.g., nausea, vomiting)	260 (8.3)	51,317 (55.8)	51,577 (54.2)	<0.001	0.720 (0.630–0.820)
Liver and biliary system	35 (1.1)	102 (0.1)	137 (0.1)	<0.001	10.188 (6.928–14.982)
Metabolic and nutritional	9 (0.3)	127 (0.1)	136 (0.1)	0.029	2.086 (1.060–4.105)
Endocrine	0 (0.0)	2 (<0.1)	2 (<0.1)	N/E^c^	N/E ^c^
General cardiovascular	44 (1.4)	59 (0.1)	103 (0.1)	<0.001	22.218 (15.012–32.882)
Myocardial, endocardial, pericardial, and valve	0 (0.0)	1 (<0.1)	1 (<0.1)	N/E ^c^	N/E ^c^
Heart rate and rhythm (e.g., hypotension)	1038 (33.1)	2650 (2.9)	3688 (3.9)	<0.001	16.724 (15.379–18.187)
Vascular (extracardiac)	11 (0.4)	72 (0.1)	83 (0.1)	<0.001	4.503 (2.385–8.501)
Respiratory system (e.g., dyspnea, decreased oxygen saturation)	608 (19.4)	995 (1.1)	1603 (1.7)	<0.001	22.046 (19.782–24.569)
Red blood cell	3 (0.1)	9 (<0.1)	12 (<0.1)	N/E ^c^	N/E ^c^
White cell and reticuloendothelial system	16 (0.5)	50 (0.1)	66 (0.1)	<0.001	9.449 (5.375–16.609)
Platelet, bleeding, and clotting	8 (0.3)	19 (<0.1)	27 (<0.1)	<0.001	12.405 (5.426–28.358)
Urinary system (e.g., nephrotoxicity)	51 (1.6)	1187 (1.3)	1238 (1.3)	0.1	1.267 (0.955–1.681)
Reproductive (male)	2 (0.1)	0 (0.0)	2 (<0.1)	N/E ^c^	N/E ^c^
Reproductive (female)	9 (0.3)	9 (<0.1)	18 (<0.1)	<0.001	29.474 (11.692–74.301)
Fetal	1 (<0.1)	1 (<0.1)	2 (<0.1)	N/E ^c^	N/E ^c^
Neonatal and infancy	4 (0.1)	9 (<0.1)	13 (<0.1)	<0.001	13.079 (4.025–42.492)
Neoplasms	0 (0.0)	1 (<0.1)	1 (<0.1)	N/E ^c^	N/E ^c^
Body as a whole—general	234 (7.5)	2748 (3.0)	2982 (3.1)	<0.001	2.634 (2.293–3.025)
Application site	3 (0.1)	402 (0.4)	405 (0.4)	N/E ^c^	N/E ^c^
Resistance mechanism	5 (0.2)	3 (<0.1)	8 (<0.1)	N/E ^c^	N/E ^c^
Secondary terms—events ^e^	2 (0.1)	6 (<0.1)	8 (<0.1)	N/E ^c^	N/E ^c^
Poison specific terms	0 (0.0)	2 (<0.1)	2 (<0.1)	N/E ^c^	N/E ^c^

Abbreviations: SOC, System Organ Class; ROR, reporting odds ratio; CI, confidence interval; N/E, not estimated. Data presented as the number of cases (% relative frequency). ^a^ *p*-value from the Mantel–Haenszel test between serious and nonserious events. ^b^ ROR from the Mantel–Haenszel test for serious events compared to nonserious events. ^c^ Statistical tests not performed due to insufficient number of reported cases (unreliable data). ^d^ Includes metallic taste, dysgeusia, and dysosmia. ^e^ Includes off-label use, drowning, accidental drug overdose, and medication errors.

**Table 4:** Univariate and multivariate analyses for the association between predictors and sedative-induced serious adverse events (AEs).

Predictors	Univariate Analysis (Number of Cases (%))			Multivariate Analysis	
	Serious AEs (n = 3132)	Nonserious AEs (n = 92,056)	*p* Value	OR (95% CI)	*p* Value
**Sex ^a^**			<0.001		<0.001
Male	1460 (46.6)	30,064 (32.7)		1.421 (1.318–1.531)	
Female	1622 (51.8)	61,176 (67.5)		1 (reference)	
**Age ^b^**			<0.001		<0.001
< 10	211 (6.7)	1661 (1.8)		1 (reference)	
10–19	130 (4.2)	4518 (4.9)		0.499 (0.388–0.642)	
20–29	164 (5.2)	7136 (7.8)		0.413 (0.323–0.528)	
30–39	249 (8.0)	10,339 (11.2)		0.427 (0.340–0.537)	
40–49	374 (11.9)	14,534 (15.8)		0.470 (0.379–0.583)	
50–59	515 (16.4)	18,228 (19.8)		0.486 (0.395–0.599)	
≥ 60	1388 (44.3)	32,319 (35.2)		0.729 (0.599–0.886)	
**Causality ^c^**			<0.001		<0.001
Certain	49 (1.6)	1773 (1.9)		1.015 (0.786–1.312)	
Probable/likely	956 (30.5)	43,258 (47.0)		0.649 (0.598–0.704)	
Possible	2127 (67.9)	47,025 (51.1)		1 (reference)	
**Etiologic sedative**			<0.001		<0.001
Alfentanil	3 (0.5)	671 (99.5)		1 (reference)	
Dexmedetomidine	66 (20.8)	251 (79.2)		35.784 (11.120–115.153)	
Diazepam	135 (5.8)	2206 (94.2)		8.384 (2.652–26.506)	
Etomidate	49 (28.2)	125 (71.8)		52.232 (15.943–171.118)	
Fentanyl	819 (1.5)	55,145 (98.5)		3.084 (0.989–9.615)	
Ketamine	148 (20.4)	578 (79.6)		31.322 (9.865–99.453)	
Midazolam	484 (10.0)	4398 (90.0)		18.174 (5.817–56.786)	
Pethidine	1019 (4.1)	23,649 (95.9)		7.861 (2.522–24.501)	
Propofol	264 (15.0)	1492 (85.0)		27.226 (8.669–85.504)	
Remifentanil	199 (6.9)	2678 (93.1)		13.207 (4.200–41.528)	
Sufentanil	2 (0.5)	405 (99.5)		0.786 (0.130–4.735)	
Thiopental	30 (7.5)	372 (92.5)		11.049 (3.287–37.148)	
**Number of concurrently used medications**			<0.001	1.015 (1.000–1.031)	<0.001
1	2062 (65.8)	68,330 (74.7)			
2	498 (15.9)	12,432 (13.6)			
3	216 (6.9)	4778 (5.2)			
4	124 (4.0)	1541 (1.7)			
≥ 5	232 (7.4)	4975 (0.8)			
**Concomitantly used medication**					
Antihistamine	67 (2.1)	649 (0.7)	<0.001	1.557 (1.096–2.212)	<0.001
NSAIDs	135 (4.3)	5605 (6.1)	<0.001	0.426 (0.343–0.527)	<0.001
Opioids	181 (5.8)	8137 (8.9)	<0.001	0.488 (0.409–0.583)	<0.001
5HT_3_ antagonists	38 (1.2)	2574 (2.8)	<0.001	0.483 (0.346–0.676)	<0.001
ASA	27 (0.9)	111 (0.1)	<0.001	2.900 (1.771–4.750)	<0.001
Neuromuscular blockers	152 (4.9)	1383 (1.5)	<0.001	1.640 (1.086–1.712)	<0.001
Corticosteroids	98 (3.1)	609 (0.7)	<0.001	2.933 (2.187–3.935)	<0.001
Sedatives	500 (16.0)	6568 (7.1)	<0.001	1.272 (1.121–1.444)	<0.001
Benzodiazepines	51 (1.6)	324 (0.4)	<0.001	2.370 (1.703–3.299)	<0.001
Zolpidem	10 (0.3)	207 (0.2)	<0.001	0.290 (0.142–0.591)	<0.001

Abbreviations: OR, odds ratio; CI, confidence interval; NSAIDs, non-steroidal anti-inflammatory drugs; 5HT, serotonin; ASA, aspirin (i.e., acetylsalicylic acid). ^a^ Information was missing in 50 (1.5%) and 816 (0.9%) serious and nonserious cases, respectively. ^b^ Information was missing in 101 (3.2%) and 3321 (3.6%) serious and nonserious cases, respectively. ^c^ Causality assessment was based on the WHO-UMC criteria.

## Data Availability

The datasets generated and/or analyzed during the current study are not publicly available due to the inclusion of private medical information but may be available from the corresponding author upon reasonable request.
